# Caffeic Acid Phenethyl Ester (CAPE) Synergistically Enhances Paclitaxel Activity in Ovarian Cancer Cells

**DOI:** 10.3390/molecules28155813

**Published:** 2023-08-01

**Authors:** Anna Kleczka, Radosław Dzik, Agata Kabała-Dzik

**Affiliations:** 1 Department of Pathology, Faculty of Pharmaceutical Sciences in Sosnowiec, Medical University of Silesia in Katowice, Ostrogórska 30, 41-200 Sosnowiec, Poland; adzik@sum.edu.pl; 2 Department of Biosensors and Processing of Biomedical Signals, Faculty of Biomedical Engineering, Silesian University of Technology, Roosevelta 40, 41-800 Zabrze, Poland; radoslaw.dzik@gmail.com

**Keywords:** propolis, caffeic acid phenethyl ester, paclitaxel, ovarian cancer, apoptosis

## Abstract

Caffeic acid phenethyl ester (CAPE) belongs to the phenols found in propolis. It has already shown strong antiproliferative, cytotoxic and pro-apoptotic activities against head and neck cancers and against breast, colorectal, lung and leukemia cancer cells. Ovarian cancer is one of the most dangerous gynecological cancers. Its treatment involves intensive chemotherapy with platinum salts and paclitaxel (PTX). The purpose of this study was to evaluate whether the combined use of CAPE and paclitaxel increases the effectiveness of chemotherapeutic agents. The experiment was performed on three ovarian cancer lines: OV7, HTB78, and CRL1572. The effect of the tested compounds was assessed using H-E staining, a wound-healing test, MTT and the cell death detection ELISAPLUS test. The experiment proved that very low doses of PTX (10 nM) showed a cytotoxic effect against all the cell lines tested. Also, the selected doses of CAPE had a cytotoxic effect on the tested ovarian cancer cells. An increase in the cytotoxic effect was observed in the OV7 line after the simultaneous administration of 10 nM PTX and 100 µM CAPE. The increase in the cytotoxicity was dependent on the CAPE dosage (50 vs. 100 µM) and on the duration of the experiment. In the other cell lines tested, the cytotoxic effect of PTX did not increase after the CAPE administration. The administration of PTX together with CAPE increased the percentage of apoptotic cells in the tested ovarian cancer cell lines. Moreover, the simultaneous administration of PTX and CAPE enhanced the anti-migration activity of the chemotherapeutic used in this study.

## 1. Introduction

Each year, approximately 240,000 women worldwide are diagnosed with ovarian cancer (OC). Ovarian cancer ranks as the second most common type of gynecological cancer and has a low survival rate. The incidence of ovarian tumors continues to increase. For every seventy women, one will develop ovarian cancer, and up to one in one hundred will die from the disease. The reason for such an unfavorable prognosis is the late diagnosis of the disease. Ovarian cancer does not give clinical symptoms for a long time, and the reported abdominal pain, fatigue and problems with urination are not specific [1,2].

The treatment of ovarian cancer involves multi-stage chemotherapy preceded by a complete surgical operation. Unfortunately, patients often relapse, even after an initial positive response. One of the most serious problems in successful cancer treatment remains the development of resistance to chemotherapy. For cancer cells, multidrug resistance is defined as the acquisition of concurrent insensitivity to several groups of different, unrelated therapeutic drugs. It usually develops in reaction to the administration of a single cytostatic drug [3]. Among the pharmacological factors, the development of drug resistance may be influenced by incorrect dosages and changes in metabolism and the bioavailability of the drug. The cellular mechanisms involved in the formation of multidrug cross-resistance include changes in the rate of drug entry into the cell and in their transport between the nucleus and the cytoplasm, the amount and affinity of target enzymes for cytostatics, the activation or inactivation of pharmacological compounds in cancer cells, the ability of cancer cells to disturb the regulation of apoptosis, changes in DNA repair processes and the possibility of removing cytostatics out of the cell by membrane transport proteins [4,5]. The mechanisms of developing drug resistance are still not fully understood, so it is important to identify new approaches to treating ovarian cancer. There is an urgent need for new, highly effective therapies for the treatment of advanced ovarian cancer [6].

The usual dose of paclitaxel used to treat ovarian cancer is 60–250 mg/m^2^ for 1–96 h and at intervals of 1–3 weeks. Nevertheless, there is no perfect strategy for the dosage of paclitaxel in gynecological cancer, even after practicing it for almost two decades. It remains a challenge for researchers to find the least-toxic, inexpensive but effective paclitaxel dosing protocol [7]. Despite the undeniable progress in the treatment of gynecological diseases, patients with ovarian cancer still experience recurrences or severe complications after chemotherapy treatment. Clinicians suggest that the discovery of a natural compound that could be administered synergistically with PTX and enhance its anti-cancer effect would reduce the toxicity of the treatment and improve the quality of patients’ lives.

Flavonoid derivatives have been proven to have antibacterial, anti-inflammatory, immunomodulatory and anesthetic properties. It has already been shown that there is a positive correspondence between a diet that contains flavonoids (mainly taken from food like vegetables, honey and fruits) and a lower risk of breast cancer [8,9,10]. There are reports of positive impacts also on colon and prostate cancers [11]. Nowadays, scientists are actively seeking natural ingredients that can be used as potential anti-cancer agents. Plant extracts are also strong candidates for the treatment of various types of cancer via a modulated apoptotic pathway. Reports in the literature also suggest that flavonoids may increase the effectiveness of routinely used chemotherapeutic agents or protect normal cells from the side effects of anti-cancer therapy [12,13,14].

Many years of experience have shown that propolis can be used alone or as an auxiliary in the treatment of many systemic diseases Propolis has antibacterial and antioxidant properties, supports immunity and accelerates tissue regeneration. Its antifungal and antiviral properties are also documented [15,16].

One of the most important components of propolis is CAPE (*caffeic acid phenethyl ester*). Caffeic acid and its natural derivatives and synthetic analogues are powerful antioxidants that protect cells from stress even in low concentrations. CAPE effectively removes reactive oxygen species (ROS) and synthetic radicals, chelates metal ions and inhibits lipid peroxidation. In addition, CAPE can affect cell metabolism and regulate inflammation, angiogenesis and apoptosis [17,18].

The main purpose of this study was to conduct an assessment of the effect of CAPE and paclitaxel administered jointly to ovarian cancer cells in in vitro studies. In the experiment, the effect of these substances on the viability and migration capacity of cells of various histological types of ovarian cancer were examined; the effect of CAPE and paclitaxel was tested on the lines of serous ovarian cancer (OV7), adenocarcinoma (HTB76) and teratocarcinoma (CRL1572). The doses of CAPE and paclitaxel that were most effective in inhibiting cell proliferation and migration were determined. In addition, it was examined whether the tested compounds induced apoptosis in ovarian cancer cells.

## 2. Results

### 2.1. Microscopic Evaluation of Ovarian Cells’ Morphology in Hematoxylin and Eosin Staining Protocol

Serum ovarian carcinoma cells of the OV7 line have a morphology that is typical of neoplastic cells. The cells are dominated by large, darkly colored nuclei with a visible nucleus. A wide cytoplasmic ridge forming long protrusions is visible around the nucleus. In the cells of the HTB76 line, attention is also drawn to the dark cell nucleus with a disentangled cytoplasm (visible subdivision figures). The cells are irregularly shaped and form clusters. Their strong intercellular adhesion is worth noting. The cell morphology of the CRL1572 cell line shows fusiform cells with long cytoplasmic protrusions. In the center of the cells, there are large, dark purple cell nuclei. Some of the cells are multinucleated.

The use of PTX at a concentration of 10 nM caused a significant number of the cells to die and detach from the slide surface. In every tested cell line, significant nucleus hyper-chromia and thinning of the cytoplasm were observed. The microscopic images also showed cell “shadows”—cells with a poorly marked nucleus and a diffuse cytoplasm containing granules. All the ovarian cancer cells were noticeably smaller; their cell nuclei were observed to vary in size, shape and color. In the OV7 and CRL1572 cell lines, the cytoplasmic protrusions were shortened.

The strongest effect of 50 µM CAPE was observed in the OV7 cell line. A significant reduction in the number of cells observed in the field of view, weakening of cell contact and a strong density of nuclei were noted. The effect of CAPE on the HTB76 cells was weaker. The condensation of cell nuclei, the shortening of cytoplasmic protrusions and the presence of pale “cell shadows” were observed in the preparation. The cells of the CRL1572 line treated with 50 µM CAPE did not significantly change their morphology. Only a reduction in the volume of the cytoplasm and its vacuolation were observed.

After using 10 nM PTX with 50 µM CAPE, the number of cells observed in the field of view decreased significantly. Cell–cell contact was weakened. The cell nuclei were condensed in all the cell lines tested, especially in the HTB76 lineage, where the cell nuclei became pyknotic. The appearance of aquatic warblers was observed in the cytoplasm of some cells.

**Figure 1:**
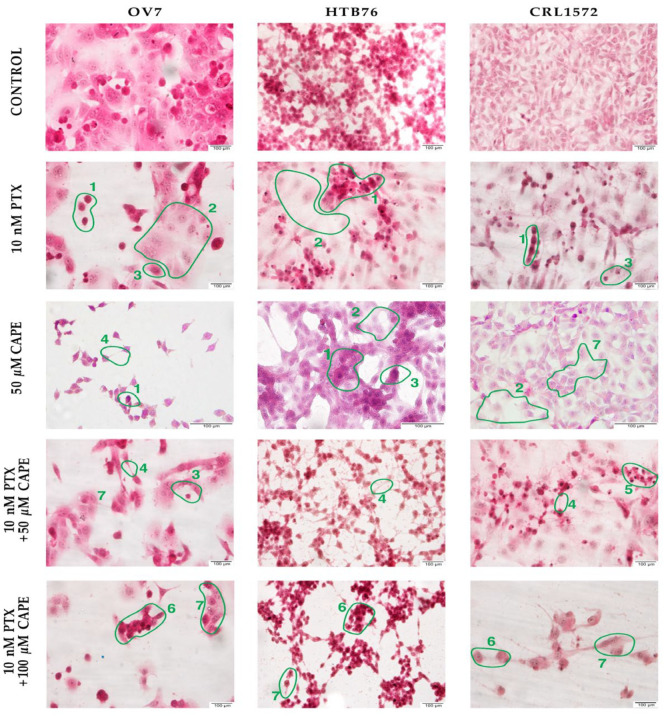
Morphological image of OV7, HTB76 and CRL1572 cells treated with 10 nM PTX, 50 µM CAPE and 10 nM PTX together with 50 µM and 100 µM CAPE after 24 h of incubation. To prepare the samples, a hematoxylin and eosin staining method was used. Exposition: optical magnification ×400. (1)—hyper-chromia, (2)—cell “shadows”, (3)—shortened cytoplasmic protrusions, (4)—weakened cell–cell contact, (5)—cell condensation and pyknotic cell nuclei, (6)—small cells, irregular cytoplasmic outline and densified nuclei, (7)—cells with irregular shape.

The administration of 10 nM PTX and 100 µM CAPE to the cell cultures did not increase the previously observed morphological changes. All the cells tested remained small with an irregular cytoplasmic outline and densified nuclei. All the tested cells retained a small size and irregular shape. The changes in the morphology of the ovarian cancer cells treated with PTX and PTX with selected CAPE concentrations are shown in Figure 1.

### 2.2. Cell Viability by MTT Assay

**Figure 2:**
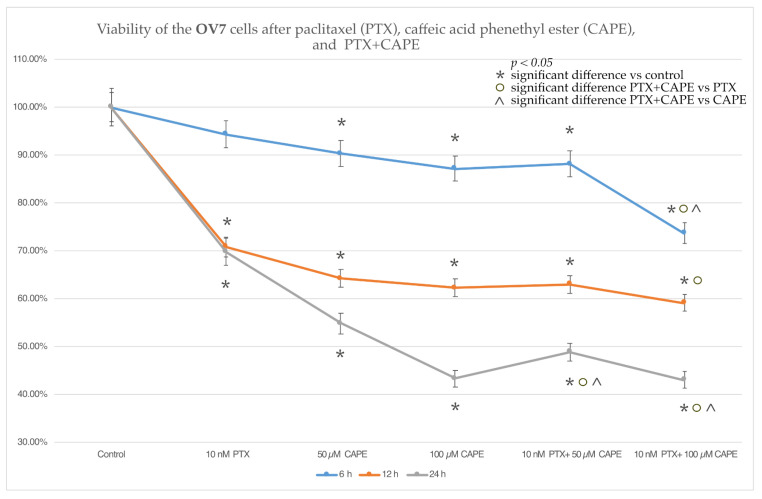
Viability of the OV7 cells exposed to 10 nM paclitaxel (PTX), 50 µM and 100 µM caffeic acid phenethyl ester (CAPE) and paclitaxel with 50 µM and 100 µM caffeic acid phenethyl ester (PTX+CAPE) treatment after 6 h, 12 h and 24 h incubation periods. Cytotoxic properties were assessed using an MTT cell viability assay. The results are presented as the mean and standard deviation of three independent experiments with twelve wells each (*p* < 0.05; * significant difference vs. control; ° significant difference in PTX vs. PTX+CAPE; ^ significant difference in CAPE vs. PTX+CAPE).

The MTT test was used to assess the viability of the ovarian cancer cells. For the OV7 line, it was shown that the application of PTX at a dose of 10 nM caused a statistically significant decrease in cell viability after a 12 h and 24 h incubation. The administration of selected doses (50 µM and 100 µM) of CAPE to the OV7 cell line resulted in a statistically significant reduction in cell viability relative to the control at each of the times tested. A significant decrease in the viability of the OV7 cells comparing to control was also observed after the administration of PTX together with 50 µM CAP. The OV7 cells treated with 10 nM PTX + 100 µM CAPE showed a significant decrease in viability for all the incubation times. Statistically significant differences in the viability of the cells treated with PTX alone and those intoxicated with PTX with a total of 100 µM CAPE were also demonstrated. In addition, it was shown that after 6 h and 24 h of incubation, the administration of 10 nM PTX+ 100 µM CAPE statistically significantly reduced the viability of the OV7 cells compared to the use of CAPE alone. The data are presented in Figure 2.

**Figure 3:**
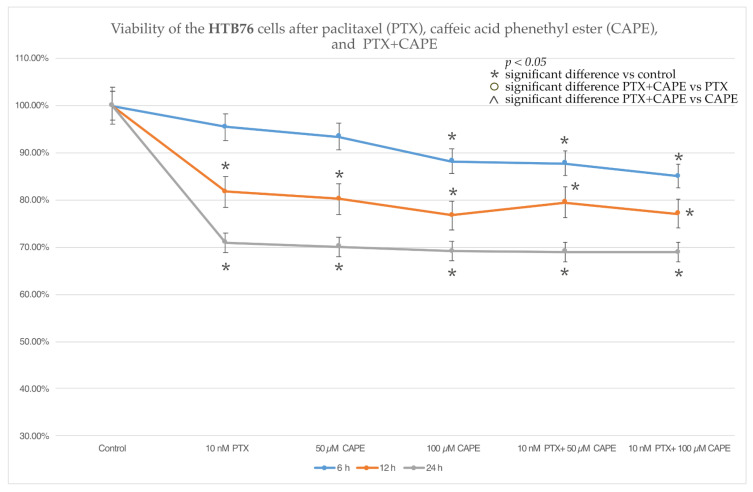
Assessment of viability of HTB76 cells exposed to 10 nM paclitaxel (10 nM PTX), 50 µM and 100 µM caffeic acid phenethyl ester (CAPE) and paclitaxel together with 50 µM and 100 µM CAPE with 6 h, 12 h and 24 h incubation periods. An MTT cell viability test was used to evaluate cytotoxicity activity. The results are presented as the mean and standard deviation of three independent experiments with twelve wells each (*p* < 0.05; * significant difference vs. control; ⁰ significant difference in PTX+CAPE vs. PTX; ^ significant difference in PTX+CAPE vs. CAPE).

The HTB76 cell line responded to the tested compounds similarly to the ovarian teratocarcinoma cells. After 12 h and 24 h, both PTX, CAPE and PTX administered together with 50 µM and 100 µM CAPE caused a statistically significant decrease in cell viability relative to the control. However, after a 6 h study, no statistically significant change was observed in the viability of the HTB76 cells exposed to 10 nM PTX and 50 µM CAPE. There was no enhancement of the cytotoxic effect of PTX after co-administration with CAPE. The data are presented in Figure 3.

**Figure 4:**
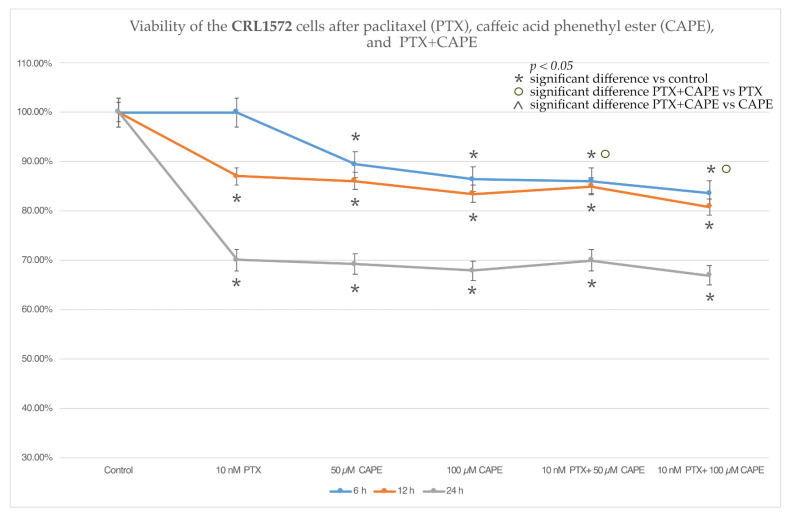
CRL1572 cell viability after 10 nM paclitaxel (PTX), 50 µM and 100 µM caffeic acid phenethyl ester (CAPE) and paclitaxel with 50 µM and 100 µM caffeic acid phenethyl ester (PTX+CAPE) treatment with 6 h, 12 h and 24 h incubation periods. CRL1572 cell viability was measured by an MTT cell viability assay. The results are presented as the mean and standard deviation of three independent experiments with twelve wells each (*p* < 0.05; * significant difference vs. control; ° significant difference in PTX+CAPE vs. PTX; ^ significant difference in PTX+CAPE vs. CAPE).

The cytotoxic effects of PTX at a dose of 10 nM on the CRL1572 cells were observed only after the 12 h and 24 h duration of the experiment. The tested doses of CAPE and the administration of PTX together with CAPE caused a statistically significant reduction in cell viability compared to the control. It was also observed that administration of CAPE did not increase the cytotoxic properties of PTX in an experiment lasting more than 6 h. After a 6 h incubation, it was noticed that the administration of PTX together with 50 µM and 100 µM CAPE significantly reduced the viability of the tested cells compared to the use of PTX alone. The described data are presented in Figure 4.

### 2.3. Assessment Apoptotic Cells by Cell Death ELISA Kit

Cell death detection ELISA is a photometric enzyme immunoassay that is used for the quantitative in vitro determination of cytoplasmic histone-associated DNA fragments (mono- and oligonucleosomes) after induced cell death. Histone-associated DNA fragments are known to be present in the cytoplasm of cells undergoing apoptosis.

**Figure 5:**
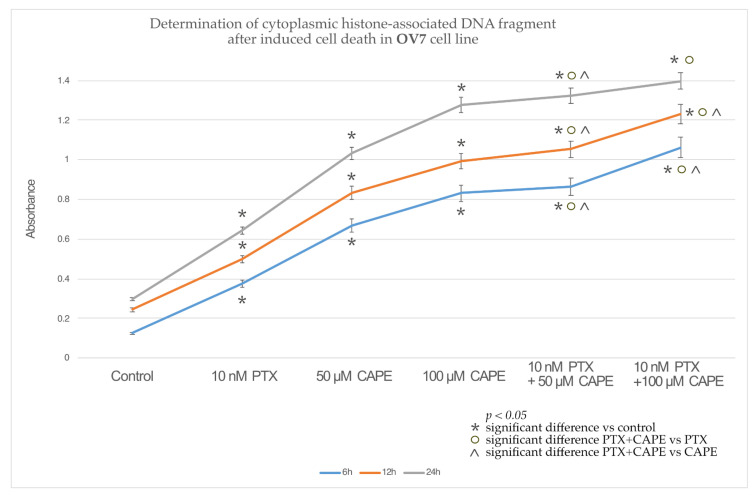
Percentage of apoptotic cells based on the concentration of histone-associated DNA fragments in the OV7 cell line treated with 10 nM paclitaxel, 50 µM and 100 µM CAPE and 10 nM paclitaxel together with 50 µM and 100 µM CAPE after 6 h, 12 h and 24 h incubation. The results are from three independent experiments. Means and standard deviations were calculated from the results of the 12 test wells (*p* < 0.05; * significant difference vs. control; ° significant difference in PTX+CAPE vs. PTX; ^ significant difference in PTX+CAPE vs. CAPE).

In the OV7 line treated with 10 nM PTX, both of the CAPE concentrations and 10 nM PTX together with 50 µM and 100 µM CAPE, at all of the times tested, a statistically significant increase in the percentage of apoptotic cells relative to the control was observed. In addition, it was shown that PTX administered together with CAPE statistically significantly increased the number of apoptotic cells compared to the culture in which the chemotherapeutic agent and CAPE were administered alone. Only within 24 h was the increased percentage of cells undergoing apoptosis not confirmed in the experiment with PTX administered together with 100 µM CAPE compared to the use of CAPE alone. The highest number of apoptotic cells was detected after a 24 h incubation of the OV7 line with the tested compounds. The described results are presented in Figure 5.

**Figure 6:**
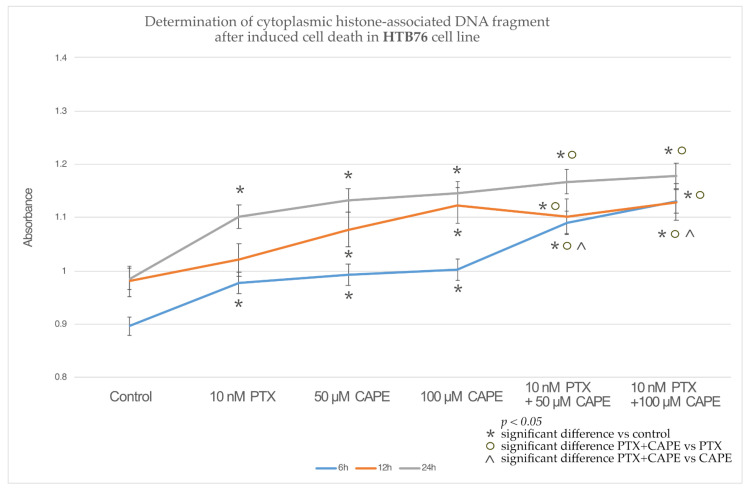
Percentage of apoptotic cells based on the concentration of histone-associated DNA fragments in the HTB76 cell line treated with 10 nM paclitaxel, 50 µM and 100 µM caffeic acid phenethyl ester (CAPE) and 10 nM paclitaxel together with 50 µM and 100 µM CAPE after 6 h, 12 h and 24 h incubation. The graph shows the results of the mean and standard deviation of three independent test repetitions with twelve wells each (*p* < 0.05; * significant difference vs. control; ° significant difference in PTX+CAPE vs. PTX; ^ significant difference in PTX+CAPE vs. CAPE).

The culture of HTB76 cells showed a statistically significant increase in the percentage of cells undergoing apoptosis compared to the control after the application of each of the tested compounds and at each time. Only PTX administered alone, after 12 h, did not induce increased apoptosis in the cells tested. It was observed that the administration of PTX together with CAPE enhanced the apoptosis of the HTB76 cells compared to the study in which the cells were only intoxicated with PTX. In addition, after 6 h of the experiment, a statistically significant increase in the number of apoptotic cells was demonstrated after the administration of PTX and CAPE in relation to the administration of CAPE alone, as shown in Figure 6.

**Figure 7:**
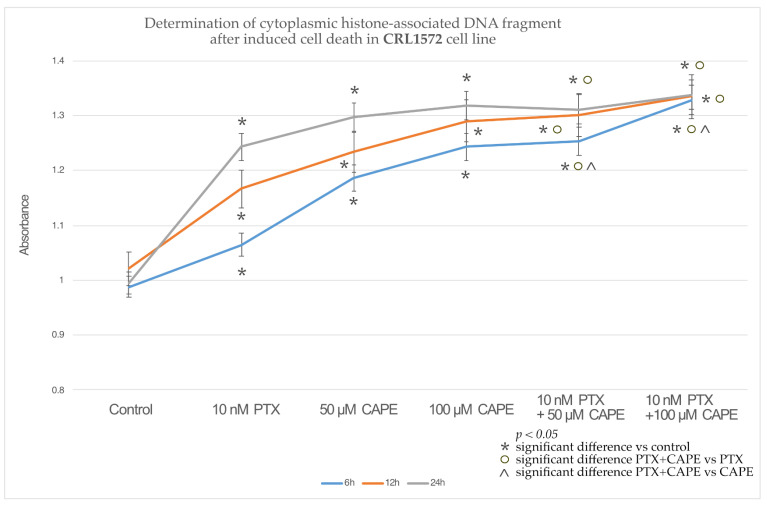
Percentage of apoptotic cells based on the concentration of histone-associated DNA fragments in the CRL1572 cell line treated with 10 nM paclitaxel, 50 µM and 100 µM caffeic acid phenethyl ester (CAPE) and 10 nM paclitaxel together with 50 µM and 100 µM CAPE after 6 h, 12 h and 24 h incubation. The mean and standard deviation from three independent experiments with twelve wells each are presented in the graph (*p* < 0.05; * significant difference vs. control; ° significant difference in PTX+CAPE vs. PTX; ^ significant difference in PTX+CAPE vs. CAPE).

Similar results were observed in the studies on the CRL1572 line. Each of the tested compounds, administered alone or in combination, increased the percentage of apoptotic cells relative to the control. The combined administration of PTX and CAPE caused an increase in apoptosis compared to the administration of only the chemotherapeutic agent. Moreover, after 6 h, the use of PTX with 50 µM and 100 µM CAPE showed a statistically significantly higher percentage of apoptotic cells than in the experiment where cells of the CRL1572 line were exposed to CAPE alone. The results are presented in Figure 7.

### 2.4. Wound-Healing

**Figure 8:**
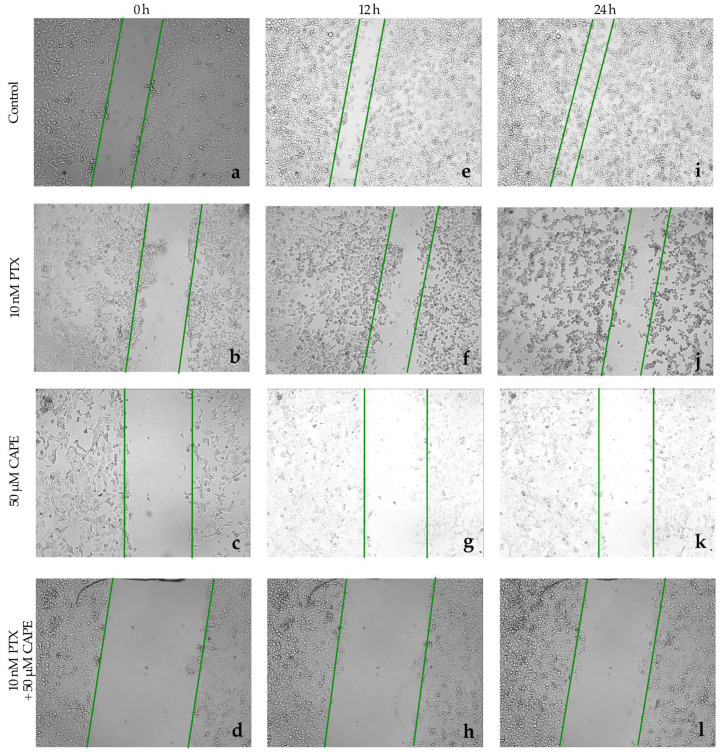
Paclitaxel (PTX), 50 µM caffeic acid phenethyl ester (CAPE) and paclitaxel with 50 µM caffeic acid phenethyl ester (PTX+CAPE) showed an anti-migration effect on OV7 cells. Pictures (**a**–**d**): scratch area of the OV7 cell line at the beginning of the experiment; (**e**–**h**): scratch area of the OV7 cell line after 12 h incubation time with test compounds; (**i**–**l**): scratch area of the OV7 line after 24 h incubation with test compounds. The cell migration factor was performed by a monolayer wound-healing assay and embedded using the free ImageJ software version 1.53t.

**Figure 9:**
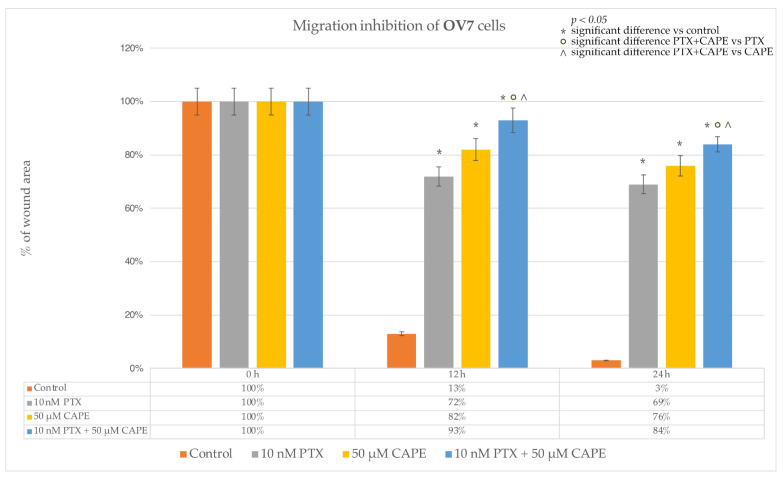
The plot of the dependence of the OV7 migration inhibition on the time and dose of the tested compounds. The results are presented as the gap area in relation to the area value of the initial scratch after 12 h and 24 h of observation. (*p* < 0.05; * significant difference vs. control; ° significant difference in PTX + CAPE vs. PTX; ^ significant difference in PTX+CAPE vs. CAPE).

The ability of paclitaxel, CAPE and paclitaxel co-administered with CAPE to inhibit the migration of neoplastic cells was investigated in a wound-healing test. In the OV7 line, to which 10 nM PTX was administered, it was observed that after 12 h, cell migration was inhibited by as much as 72%. The ovarian cancer cells did not overgrow the gap that had been formed. After extending the test time to 24 h, it was observed that cell migration was inhibited in 69%. Single cells began to overlap the edges of the wound. After the application of 50 µM CAPE in the cell culture, an even stronger anti-migration effect was observed. After 12 h of incubation, it was noticed that 82% of the initial wound area had not healed. After extending the test time to 24 h, the surface area of the scratch was occupied by migrating cells in 76%. After the use of 10 nM PTX in the culture together with a dose of 50 µM CAPE, the inhibition of cell migration after 12 h was over 90%, and after 24 h it was maintained at 84%. The simultaneous administration of PTX and CAPE resulted in a statistically significantly greater inhibition of OV7 cell migration compared to the effect shown for the single administration of the tested compounds. Microscopic images of the created wound and the process of its overgrowing by the cells of the OV7 line are presented in Figure 8. Figure 9 shows a graph of the inhibition of migration in the studied line.

**Figure 10:**
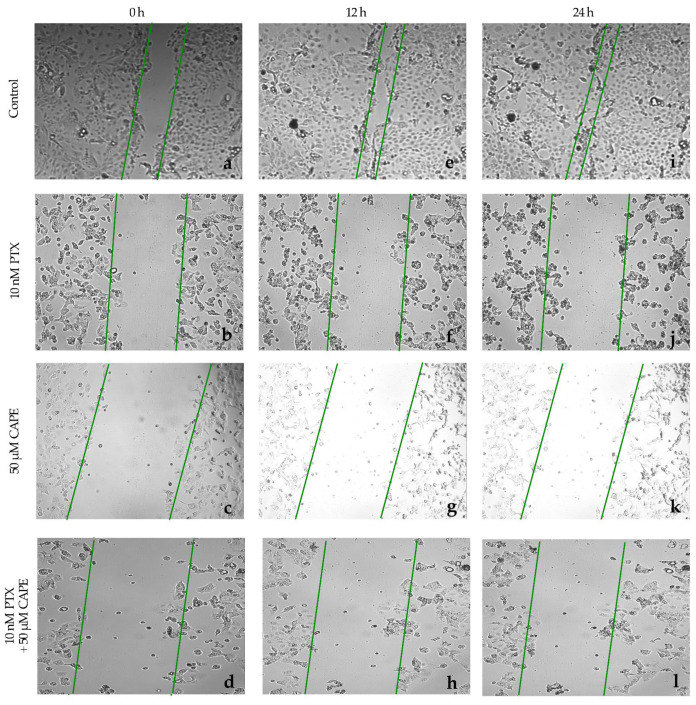
Paclitaxel (PTX), 50 µM caffeic acid phenethyl ester (CAPE) and paclitaxel with 50 µM caffeic acid phenethyl ester (PTX+CAPE) promoted an inhibitory migration effect on HTB76 cells. Pictures (**a**–**d**): scratch area of the HTB76 cell line at the beginning of the experiment; (**e**–**h**): scratch area of the HTB76 cell line after 12 h incubation time with test compounds; (**i**–**l**): scratch area of the HTB76 line after 24 h incubation with test compounds The cell migration factor determination was performed with a monolayer gap-closure migration assay and embedded using the free ImageJ software version 1.53t.

**Figure 11:**
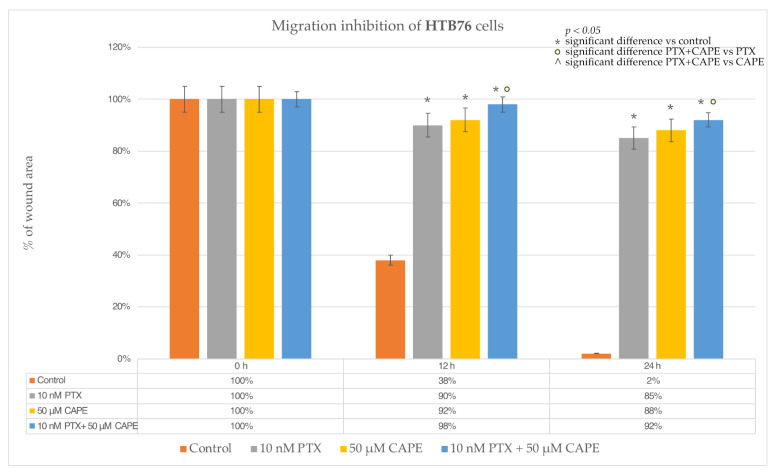
The plot of the dependence of the HTB76 migration inhibition on the time and dose of the tested compounds. The results are presented as the gap area in relation to the area value of the initial scratch after 12 h and 24 h of observation. (*p* < 0.05; * significant difference vs. control; ° significant difference in PTX + CAPE vs. PTX; ^ significant difference in PTX+CAPE vs. CAPE).

The evaluation of cell migration in the HTB76 line treated with the tested compounds showed that the dose of 10 nM PTX strongly inhibited the wound ingrowth after both 12 h and 24 h of incubation. A similar effect was observed after the administration of 50 µM CAPE; about 90% of the initial wound surface did not heal. After the simultaneous administration of 10 nM PTX and 50 µM CAPE to the culture, cell migration was almost completely stopped. The percentage of the inhibition of migration after 12 h of the experiment was 98% and remained above 90% after 24 h. For the HTB96 line, a statistically significantly stronger inhibition of cell migration was demonstrated after the administration of PTX and CAPE compared to that under the influence of PTX alone. However, no enhanced anti-proliferative effect was demonstrated with PTX+CAPE compared to CAPE alone. The described results are presented in Figure 10 and Figure 11.

**Figure 12:**
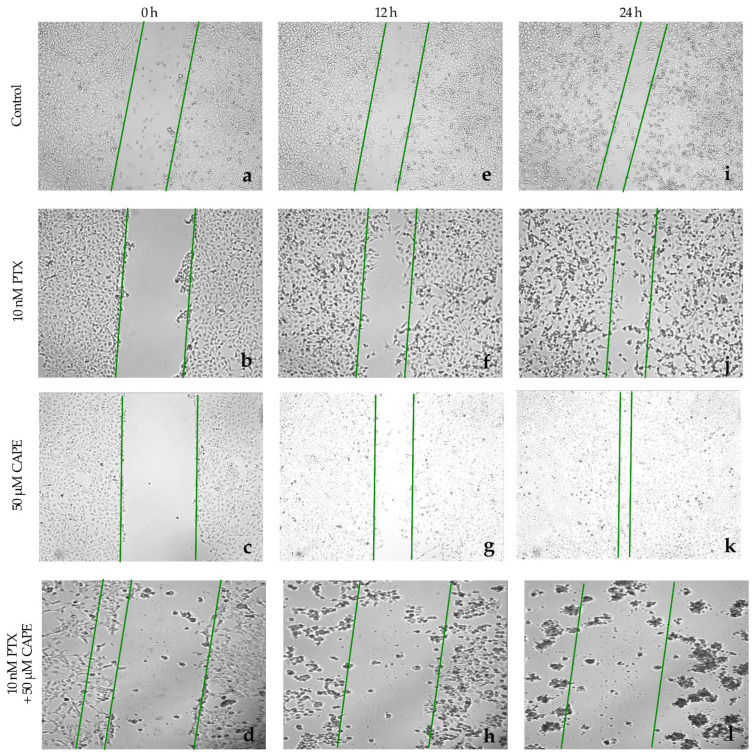
Paclitaxel (PTX), 50 µM caffeic acid phenethyl ester (CAPE) and paclitaxel with 50 µM caffeic acid phenethyl ester (PTX+CAPE) promoted an inhibitory migration effect on CRL1572 cells. Pictures (**a**–**d**): scratch area of the CRL1572 cell line at the beginning of the experiment; (**e**–**h**): scratch area of the CRL1572 cell line after 12 h incubation time with test compounds; (**i**–**l**): scratch area of the CRL1572 line after 24 h incubation with test compounds. The cell migration factor was performed by a wound-closure test and embedded using the ImageJ software version 1.53t.

**Figure 13:**
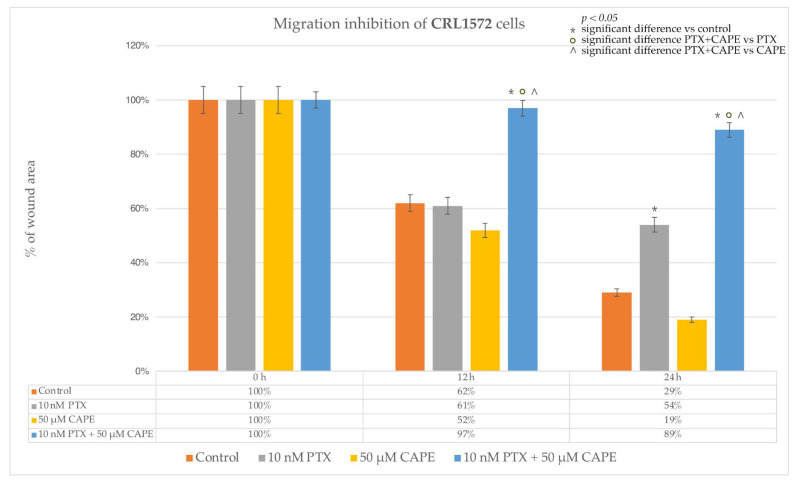
The plot of the dependence of the CRL1572 migration inhibition on the time and dose of the tested compounds. The results are presented as the gap area in relation to the area value of the initial scratch after 12 h and 24 h of observation. (*p* < 0.05; * significant difference vs. control; ° significant difference in PTX + CAPE vs. PTX; ^ significant difference in PTX+CAPE vs. CAPE).

An interesting dependence was observed in the CRL1572 line. PTX administered to the culture at a dose of 10 nM for 12 h did not stop the cell migration; it was comparable to the cell migration observed in the control culture. Only after extending the duration of the experiment to 24 h was it observed that the inhibition of the migration of the cells treated with PTX was over 50% and significantly higher than in the control. The administration of 50 µM CAPE also did not result in a statistically significant inhibition of migration relative to the control. After both 12 h and 24 h of the experiment, the scratch surface area was comparable in the control culture and in the culture treated with the selected dose of CAPE. The administration of PTX together with 50 µM CAPE to the CRL1572 line turned out to be much more effective. These substances almost completely inhibited the cell movement after 12 h. The anti-migratory effect was maintained after the 24 h duration of the experiment. Figure 12 shows the process of wound healing in the tested line, while Figure 13 shows a graph of the inhibition of cell migration depending on the time and concentration of the tested compounds.

## 3. Discussion

Paclitaxel is one of the most effective anti-cancer drugs. Paclitaxel inhibits cell division. The process of mitotic cell division requires the formation of fibrous protein structures, so-called microtubules, which are made of smaller subunits, i.e., the tubulin protein. It binds to proteins involved in microtubule formation and promotes the formation of microtubules and stabilizes them by inhibiting their decomposition. In this way, it prevents the reorganization of the microtubules, which stops the mitotic process at some point. In the cytoplasm of a cell, an abnormal system of microtubules is formed, thus disturbing the vital functions of the cell. Consequently, cell death occurs [19].

Paclitaxel is used to treat, among others, breast cancer, the late clinical stages of some histological types of lung cancer and AIDS-related Kaposi’s sarcoma [20]. Together with platinum derivatives, it is the first-line chemotherapy drug for ovarian cancer.

Almost all of the chemically synthesized drugs currently used to treat cancer have a significant toxicity to normal cells; however, various naturally occurring flavonoids have been shown to have a selective cytotoxicity in human tumor cell types with minimal toxicity to normal cells. Propolis (bee glue) is a natural apitherapeutic product rich in phenolic compounds. Flavonoids as well as phenolic acids and their esters determine the beneficial properties of propolis [21].

In numerous studies, it has been shown that natural compounds, e.g., quercetin (Que), apigenin (Api), kaempferol (Kmp), myricetin (Myr) and caffeic acid phenethyl ester (CAPE), exhibit anti-cancer properties. The above-mentioned compounds possess significant antioxidant activities [22,23,24,25,26,27,28].

The latter compound mentioned above, i.e., caffeic acid phenethyl ester (CAPE), possesses antioxidant, antibacterial, anti-inflammatory and immunomodulatory as well as anti-cancer activities [29,30,31]. CAPE has been shown to have an antiproliferative and pro-apoptotic effect on neoplastic cells in cases of breast cancer, colon cancer and lung cancer [21,28]. A previous experiment on the ovarian cancer cell line OV7 showed that CAPE induced a dose-dependent and duration-dependent cytotoxic effect in tumor cells. CAPE has been observed to induce microscopic and molecular changes characteristic of apoptosis in OV7 cells. The activity of the pro-apoptotic Bax gene was increased after the use of low (25 µM) doses of CAPE [32]. Liu et al., also showed that CAPE restrains the invasion and migration of SKOV-3 cells and inhibits their survival. Moreover, in animal experiments, the authors proved that CAPE can obstruct the growth of ovarian cancer in vivo. It was indicated that the expressions of proliferation markers (Ki67 and PCNA) were vastly repressed in the CAPE group compared with the control group [33,34].

Based on the promising reports in the literature, this experiment was designed to evaluate the effects of CAPE administered together with PTX on cells of various histological types of ovarian cancer. It was investigated whether CAPE enhances the cytotoxic activity of low doses of the routinely used chemotherapeutic agent and whether ovarian cancer cells treated with test compounds would undergo apoptosis. Moreover, the ability of PTX and CAPE to inhibit cellular migration was assessed.

The experiment was carried out on three histological types of ovarian cancer. The OV7 line is an undifferentiated, serous cancer that was collected from a patient in stage III of the neoplastic disease who was previously treated with chlorambucil. It is the most common type of ovarian cancer and has a very poor prognosis. HTB76 is an adenocarcinoma of the ovary. Ovarian adenocarcinomas account for <5% of all ovarian malignancies. When compared with their serous counterparts, a greater proportion of adenocarcinoma tumors present as early-stage (I–II) tumors and are often associated with a large pelvic mass, which may account for their earlier diagnosis and better prognosis. The CRL1572 line used in this study was established from cells taken from ascitic fluid from a girl with ovarian teratocarcinoma—a rare type of tumor derived from germ cells. Each histological type of ovarian cancer is characterized by its own pathogenesis, clinical symptoms, treatment susceptibility and prognosis [35,36].

The effect of CAPE, PTX and PTX administered together with selected doses of CAPE on the cell morphology of all the ovarian cancer lines tested was similar. The 50 µM CAPE dose had the strongest effect on the morphology of the OV7 cell line. There was a noticeable decrease in the number of cells visible in the preparation, the cell nuclei were condensed and the cytoplasmic projections were shortened. The cell lines HTB76 and CRL1572 also developed nucleus condensation and cytoplasmic granules, but the cells continued to form nests. After the use of the chemotherapeutic agent, the number of cells in the preparation decreased, the cytoplasmic protrusions were shortened and the cell nuclei—large and intertwined in the control samples—became rather pycnotic. The cytotoxic effect of PTX increased after the addition of 50 µM CAPE. The administration of 100 µM CAPE, however, did not cause any increase in the apparent cell damage. The described changes in the morphology of the cancer cells exposed to the cytotoxic substances were very typical and resembled the image of a mitotic catastrophe. Already in 1994, Rasbridge et al. [37] reported that breast cancer cells under the influence of chemotherapeutic agents become enlarged with a vacuolated cytoplasm and vesicular nuclei containing prominent nuclei; occasionally, the nuclei are angular and hyperchromatic [30]. Other features of damaged neoplastic cells including cell swelling, cytoplasmic blebs, villous projections and vacuolization were also described by Jesneko et al. [38]. The morphology of ovarian cancer cells undergoing chemotherapeutic treatment was also thoroughly investigated in 3D distributions of cellular parameters using a 3D Explorer holographic tomographic microscope in the article by Zhikhoreva et al. The authors conducted a comparative analysis of the antitumor efficacy of two cytostatics, cisplatin and dioxadet, stating an essential difference in the morphology of the cells, both normal (erythrocytes) and cancerous, present in ascitic fluid. Round-shaped carcinoma cells with a prominent internal structure were described. “Giant” cancer cells revealed in the authors’ research could be considered as dead due to mitotic catastrophe [39].

The results of determining the viability of the ovarian cancer cells in the MTT test showed that in the case of the serous ovarian cancer OV7 line, CAPE at a dose of 100 µM increased the cytotoxic effect of PTX. A dose of 50 µM CAPE enhanced the cytotoxic effect of PTX only after the 24 h duration of the experiment. For the HTB76 line, no statistically significant reduction in viability was demonstrated after the simultaneous administration of PTX and CAPE. On the other hand, in the CRL1572 line, an increase in cytotoxicity was observed after the administration of PTX and CAPE at both concentrations, but only within 6 h after the start of the experiment. The lack of a cytotoxic effect observed in the HTB76 line may have been due to the short duration of the experiment. In the treatment of ovarian cancer, PTX is administered as a 3 h or 24 h intravenous infusion; the cytotoxic effects appear in the following days, but the metabolic distribution of paclitaxel in humans is not fully understood. It is worth noting that the statistically significant enhancement of the cytotoxicity of PTX administered together with CAPE, in relation to the CRL1572 cell line after 6 h of the experiment, can be explained by the lack of any cytotoxic activity of the chemotherapeutic administered alone in the initial stage of the experiment. After 6 h from the application of 10 nM PTX to the tested cell line, no reduction in cell viability was observed. However, a statistically significant decrease in viability was demonstrated after the administration of each of the tested doses of CAPE, which suggests that the cytotoxic effect observed after the simultaneous use of both tested compounds was based on the effect of the CAPE rather than the cytotoxic activity of the chemotherapeutic agent. Moreover, the in vivo administration of PTX is almost always accompanied by an infusion of cisplatin. The simultaneous use of a combination of these chemotherapeutics is considered to be the “gold standard” of first-line ovarian cancer treatment [40]. In addition, it is worth remembering that each histological type of ovarian cancer has a different pathogenesis and tissue origin. Recent reports suggest that the histogenesis of serous ovarian cancer, adenocarcinoma and teratocarcinoma are completely different [41,42]. In ovarian adenocarcinoma, the OncoScan microarray technology is used to discover new molecular pathways for targeted therapies because in many cases adenocarcinoma chemotherapy is ineffective [43]. On the other hand, teratocarcinoma is a rare disease with limited data to guide treatment choices [44]. There is a lack of data in the literature assessing the effect of PTX and CAPE on these cell lines.

However, the use of flavonoids synergistically for routine pharmacotherapy has already been explored for other cell lines. On the example of A549 non-small-cell lung cancer cells, Klimaszewska-Wiśniewska et al., proved that the administration of fistein together with paclitaxel leads to a mitotic catastrophe in cancer cells. An MTT test showed a statistically significant reduction in the cell viability of the A549 line after the synergistic effect of low (10–50 µM) doses of fistein and doses of paclitaxel (0.1–0.5 µM), which are considered as clinically achievable. Using cytometric methods and transmission electron microscopy, it was also shown that morphological changes characteristic of autophagy and/or mitotic catastrophe occur in cells treated with the tested compounds [45].

On the example of breast cancer cell lines, it was also possible to confirm the beneficial effect of flavonoids on the effectiveness of treatment with paclitaxel and doxorubicin. Luteoil and glabridin, by suppressing Nrf2-mediated signaling and blocking STAT3, potentiated the effect of chemotherapeutics in the MDA-MB231 line [46,47]. Similar effects were also shown by quercetin and curcumin on the MCF7 cell line. The administration of compounds of a natural origin improved the permeability of the chemotherapeutics into the cells and intensified apoptosis. In addition, quercetin has been observed to inhibit the toxic effects of paclitaxel on healthy tissues [48,49,50,51,52].

Epigallocatechin gallate (EGCG), a flavonoid that is the ester of epigallocatechin and gallic acid, has also been shown to be effective in sensitizing colorectal cancer cells to the chemotherapeutic agent 5-fluorouracil. La et al., showed the inhibition of the NFκβ pathway, enhanced DNA fragmentation and an increase in the number of apoptotic cells in the tested cell lines after the simultaneous application of a dose of 50 µM EGCG [53].

An assessment of the percentage of cells that underwent apoptosis was carried out using a cell death ELISA, which quantitates the histone-associated DNA fragments characteristic of apoptotic-body-forming cells. It was shown that the combination of PTX and selected CAPE concentrations significantly increased the concentration of cytoplasmic histone-associated DNA fragments in every tested cell line. A higher concentration of CAPE and a longer duration of incubation increased the pro-apoptotic effect. A statistically significant increase in the percentage of apoptotic cells after the simultaneous administration of PTX and CAPE, compared to the results obtained after the treatment with the flavonoid alone, was most clearly observed in the OV7 cell line. In the HTB76 and CRL1572 lines, the increased pro-apoptotic activity of PTX and CAPE, compared to the administration of CAPE alone, was only statistically significant during the 6 h duration of the experiment.

CAPE is known for its pro-apoptotic properties. It causes the apoptosis of cancer cells primarily by inhibiting nuclear factor kappa B (NFκβ) and via the direct activation of caspase 3 [54,55]. Our previous studies have shown that by also regulating the BAX/BCL2 genes, CAPE can influence the severity of apoptosis in serum ovarian cancer [32]. It turns out that CAPE can also increase the percentage of apoptotic cells in cells that have been treated with the cytostatic. Paclitaxel is known as a chemotherapy drug that can suppress the proliferation of cancer cells by inducing cell cycle arrest and via the induction of mitotic catastrophe. There have been reports showing that paclitaxel may also induce apoptosis via inducing anti-tumor immunity [56]. In an experiment, it was possible to stimulate the pro-apoptotic properties of the chemotherapeutic without the participation of the cells of the immune system [57].

In this experiment, the ability of PTX, CAPE and PTX + CAPE to inhibit the migration of neoplastic cells was also tested. Using a JuliBr recorder, it was shown that the anti-migratory effect of PTX was intensified after the addition of 50 µM CAPE. The inhibitory effect of the overgrowth of the wounds was particularly evident after the 12 h experiment time. Many reports in the literature confirm these results. Wu et al., described that CAPE suppresses vascular formation by inhibiting VEGF and has the potential to suppress tumor invasion and metastasis via MMPs [58].

The role of CAPE as an inhibitor of angiogenesis and tumor metastasis and invasion has been demonstrated both in vitro and in vivo. In a study on prostate cancer, it was proven that CAPE treatment suppresses the migration and invasion of PC-3 and DU-145 PCa cells via the elevation of ROR2 (*tyrosine-kinase-like orphan receptor 2*) and E-cadherin proteins and via the reduction in vimentin protein [59]. Moreover, in a nude mouse model, the administration of CAPE inhibited the growth of LNCaP prostate cancer xenografts [60]. An inhibition of cell migration activity was also observed in examples of head and neck tumors. Chiang et al. [61] described the suppression of cell proliferation and the invasion of human nasopharyngeal cancer cells via the upregulation of NDRG1 (N-myc downstream regulated genes that belong to a family of cytosolic proteins) associated with the suppression of cyclin E protein. In the research of Kuo et al. [62], in turn, it was shown that CAPE suppresses the proliferation and survival of TW2.6 human oral squamous cell carcinoma via the inhibition of Akt signaling.

The most important features of a malignant neoplasm include the ability to invade and destroy the surrounding tissues and a tendency to metastasize to lymph nodes and distant organs. Diffuse neoplastic disease cannot be cured radically; it is only possible to slow down its progress, extend the patient’s life and maintain a good quality of life. This is why it is so important to understand the mechanisms of metastasis [63]. Limiting cellular migration could lower the risk of distant metastases and improve patients’ prognoses, but it should be remembered that serum ovarian cancer is most often diagnosed in women in the late clinical stage, when the metastases are already in the liver, lungs and bones. The inhibition of cell migration by CAPE in adenocarcinoma and teratocarcinoma lines seems to be of greater importance. In tumors that are detected quickly, the inhibition of migration may reduce the size of the tumor and reduce the risk of metastasis via the blood and lymphatic pathways [64,65,66].

Despite the undeniable advances in medicine, the early diagnosis and treatment of ovarian cancer remain a serious problem [67]. Some synthetic chemical agents that have been tested in clinical trials have failed due to their adverse effects. However, some natural products can increase the effectiveness of anti-cancer drugs and minimize the side effects of chemotherapy [68,69]. The use of CAPE in combination with paclitaxel may significantly increase therapeutic effectiveness and reduce toxicity and, last but not least, increase the compliance of patients undergoing the treatment of serous ovarian cancer.

## 4. Materials and Methods

### 4.1. Cell Lines and Reagents

#### 4.1.1. Ovarian Cancer Cell Lines

Three ovarian cancer cell lines from the ATCC collection were used in the experiment: OV7 line (stage III, mixed tumor type, poorly differentiated, ref. no.: 96020764), HTB76 (grade III adenocarcinoma) and CRL1572 (ovary teratocarcinoma, taken from ascitic fluid). The provider of the cell lines was Merck (Warsaw, Poland). Cell cultures were performed under the conditions recommended by the supplier.

The OV7 and CRL1572 cell cultures were carried out at 37 °C and whilst maintaining an atmosphere of 5% CO_2_ via the use of an MCO-170AICUV incubator (Panasonic, Oizumi-Machi, Japan). The OV7 cells were cultured on a DMEM: HAMS F12 (1:1) medium (catalog nr. D6421), whereas the CRL1572 cells were cultured in Eagle’s minimum essential medium (EMEM) (catalog no.: 30-2003). A 5% bovine serum (FBS; fetal bovine serum; PAA Laboratories, Pasching, Austria) was added to both mediums. The OV7 medium was also treated with 2 mM glutamine, 10 µg/mL insulin and hydrocortisone at a concentration of 0.5 µg/mL. The mix of antibiotics was added to both mediums at the following concentrations: 100 IU/mL of penicillin, 100 µL/mL of streptomycin and 250 µL/L of amphotericin B. The base medium for the HTB76 cell line was ATCC-formulated Leibovitz’s L-15 Medium, (catalog no.: 30-2008). To obtain the complete growth medium, fetal bovine serum was added to a final concentration of 20%. Because the L-15 medium formulation was devised for use in a free gas exchange with atmospheric air, the HTB76 cell line culture was carried out at 37 °C in a 100% air atmosphere. The cells were grown in bottles with a surface area of 25 cm^2^ and were passaged when they reached 80% confluence.

#### 4.1.2. CAPE

CAPE of ≥97% purity that was soluble in DMSO (dimethyl sulfoxide; from POCh, Poland) or ethanol, a compound by Sigma Aldrich (Warsaw, Poland, ref. No: C8221), was used in this study according to the manufacturer’s protocol.

#### 4.1.3. Paclitaxel

Paclitaxel (from *Taxus brevifolia*, ≥95% (HPLC) catalog no.: T74020) was purchased from Merck, Warsaw, Poland. In the experiment, the paclitaxel was dissolved in DMSO; before the beginning of the actual experiment, it was established that the volume of DMSO used to dissolve the reagents was not toxic to the tested cell lines.

### 4.2. Hematoxyin–Eosin Staining

After trypsinization, all the cells were inoculated onto four-chamber culture slides (Nunc Lab-Tek II Chamber Slide System, Thermo Fisher, ref. 154526PK, Rochester, NY, USA) at 1000 cells/well. A volume of 1 mL of dedicated medium was poured into each chamber. After a 24 h incubation period, when the cells clumped on the surface of the slide, the confluence was assessed at 80%.

The proper concentrations of PTX (10 nM) and CAPE+PTX (10 nM PTX + 50 µM CAPE and 10 nM PTX + 100 µM CAPE) were prepared in fresh, dedicated culture medium and put into each of the chambers. The cell culture was carried out for the next 24 h in standard conditions. Then, the chambers were disassembled, and the cells were fixed to the surface of the slide by a 12 h incubation in 96% ethyl alcohol. Next, the staining was conducted according to the protocol developed in the Histopathology Laboratory of the Department of Pathology. A decreasing ethanol series (at concentrations of 99.6%, 96%, 90%, 80%, 70% and 50%) was gradually used for cell hydration and for better dye binding, and thereafter the material was stained in a hematoxylin solution for 12 min followed by washing the slides under tap water until the blue coloring was visible. Next, the cells were incubated in eosin solution for 30 s and were then washed with PBS solution. An increasing ethanol series (50%, 70%, 80%, 90%, 96% and 99.6%) was prepared for dehydrating the cells, and thereafter the slides were incubated with a xylene mixture (50:50) for 1 min. Ultimately, a 1 h pure xylene bath of the slides was performed. The slides were closed with a Canadian lotion. To obtain the images of the morphology of the cultures, an Axiostar microscope was used (Carl Zeiss, Jena, Germany).

### 4.3. MTT Assay

An MTT assay is a common method for determining cell viability and evaluating the cytotoxic properties of test compounds; in this study, PTX and PTX administered with CAPE were evaluated. To evaluate the metabolic activity of ovarian cancer cells, the colorimetric activity was used. The principle of the MTT method is based on the assumption that tetrazolium salts in undamaged mitochondria are converted into insoluble formazan, and therefore the amount of formed formazan was counted, as it was proportional to the number of living cells. Using our experience in working with cell cultures and relying on the recommendations of the reagent supplier (Merck, Warsaw, Poland), in order to evaluate the cytotoxic properties of the tested compounds, cells of the examined lines were plated on 96-well plates at the amount of 10,000 cells per well. A total of 0.1 mL of dedicated culture medium was added to each well. In the next 24 h, the cells were left to attach to the surface of the plates. After that period, the culture medium was decanted, and PTX (10 nM) and PTX with CAPE (concentrations: 10 nM PTX + 50 µM CAPE and 10 nM PTX + 100 µM) were added to the defined wells, respectively. The culturing was continued for 6 h, 12 h and 24 h, and then the culture medium was poured out, and 10 mL of MTT (Biotium, Fremont, CA, USA) was added to each well. The incubation of the cells with the MTT reagent was continued for the next four hours. After the lapse of this period, 200 mL DMSO was added to each well to dissolve the formed formazan crystals. The measurement of the absorbance was performed by using an EL × 800 microplate reader (BioTek, Shoreline, WA, USA) with a 570 nm wavelength. In order to deduce the number of dead cells, an appropriate formula was used [69]:

(dead cells) = (1 − [absorbance of examined sample/control absorbance]) × 100%

### 4.4. Cell Death Detection ELISA

A cell death detection ELISA (catalogue no.: 11544675001 Roche, purchased from Merck, Warsaw, Poland) serves as a photometric enzyme immunoassay for the qualitative and quantitative in vitro determination of cytoplasmic histone-associated DNA fragments (mono- and oligonucleosomes) after induced cell death. This is a quantitative test that uses a sandwich enzyme immunoassay. The mouse monoclonal antibodies used in the assay indicate specific binding to DNA fragments and histones formed during apoptotic cell nucleus disintegration. This allowed the specific determination of mono- and oligonucleosomes in the cytoplasmatic fraction of the cell lysate. Anti-histone antibody reacts with the histones H1, H2A, H2B, H3 and H4 of various species (e.g., human, mouse, rat, hamster, cow, opossum and *Xenopus*). The anti-DNA-POD antibody binds to ss- and dsDNA. Therefore, the apoptotic cell death was measured in this study by ELISA via the detection of mono- and oligonucleosomes.

Using the recommendations described by the manufacturer of the assay, the tested lines were plated in 96-well plates and incubated in dedicated growth media until 80% confluence was reached. Next, the following solutions of the test compounds in the growth medium were prepared: 10 nM PTX and 10 nM PTX + 50 µM CAPE and 10 nM PTX + 100 µM CAPE; these were then added to the cell cultures. The cells were incubated for 6 h, 12 h and 24 h, and then the supernatants were prepared. For this purpose, based on the instructions, the cells from plates were shortly trypsinized and centrifuged at 200× *g* for 5 min. The supernatants were then discarded, and the cell pellets were carefully resuspended in 1 mL of dedicated culture mediums. Again, the samples were centrifuged at 200× *g*, and after 5 min, 500 μL of incubation buffer was added to each tube with cell pellets. The cell lysis lasted for half an hour, and the samples were stored at room temperature. The lysates were then centrifuged at 20,000× *g* for 10 min, and 400 μL of the obtained supernatants (containing cytoplasmic fraction) were taken straight to the measurements. Cell nuclei containing high-molecular-weight and unfragmented DNA were left in the pellet and had no effect on the experimental results. The supernatant was prediluted with incubation buffer to a ratio of 1:10 (1 × 10^4^ cell equivalents/mL; the number of cells was determined in a Burker chamber) and the nucleosomes were detected in the sample by immunoassay.

The test required the ELISA plate to be coated with 100 μL of coating solution, which was pipetted into each well of the microplate modules. Then the coated plate was covered tightly with a self-adhesive cover foil and incubated overnight at +2 °C. The next day, the coating solution was removed, and 200 μL of incubation buffer was pipetted into each well of the microplate modules. The incubation lasted 30 min at room temperature. The plates were then carefully rinsed three times with 250 μL of washing solution per well, and 100 μL of the sample solution containing the tested supernatants was added to each well. After 90 min of incubation at room temperature, the samples were decanted, and the microplates were rinsed three times with from 250 to 300 μL of washing solution per well. In the next step, 100 µL of conjugate solution was added to each well, and the plates were again incubated for approximately 90 min at room temperature. After rinsing the microplates three times again, 100 μL of substrate solution was added. After a final incubation on a plate shaker at 250 rpm (until color developed), the reading was performed at a wavelength of 405 nm [70,71].

### 4.5. Wound-Healing Assay

To measure the ability of PTX and PTX + CAPE to inhibit migration, a cell-wound-closure assay was used. This method is commonly used in our department to assess cell proliferation and mobility. Briefly, all the tested cell lines were plated in 6-well plates for no longer than 48 h to achieve an 80% confluence. Then, using a p200 pipette tip, the surface of the couture was scratched to achieve a wound—a gap. Each well was then gently washed with clean medium. After that, the process of incubation started with dedicated mediums containing 10 nM PTX and 10 nM PTX + 50 µM CAPE, respectively. The control samples were cultured without any treatment; however, to minimize the effect of proliferation, the experiments were also repeated in primary cells that had been previously treated with mitomycin-C (Bedford Laboratories, Bedford, OH, USA) at concentrations ranging from 5–10 μg/mL for 3 h. The migration rate was assessed by a monolayer gap-closure migration assay. The area of the scratch was calculated by image analysis with the ImageJ software (version 1.53t, National Institute of Health, Bethesda, MD, USA) with a wound healing tool plugin (Montpellier RIO Imaging, CNRS, Montpellier, France). Firstly, the initial area of the wound was measured and taken as a control group, and then the respective areas were measured after 12 h and 24 h. The migration inhibition was evaluated as the wound area value normalized to the initial one [72].

### 4.6. Statistical Analysis

**Figure 14:**
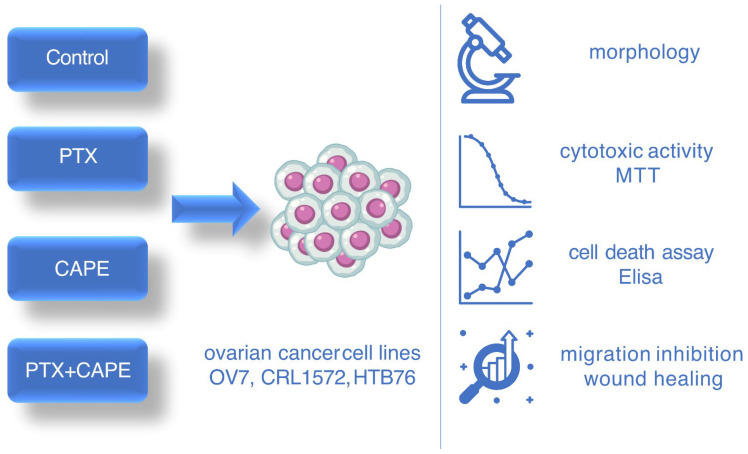
Schematic methodology of the experiment.

All the experiments were conducted three times and performed in quadruplicate (*n* = 12). The results were quoted as means ± SD and performed as independent sample *t*-tests. The respective means of the results of the tested compounds were compared with values of the control cells results. To assess the significance of the differences in the obtained results between the 6 h, 12 h, 24 h and control samples, the Friedman ANOVA test was used. All *p*-values less than 0.05 were considered statistically significant. The schematic methodology is presented in Figure 14.

## 5. Conclusions

This experiment showed that CAPE, when co-administered with paclitaxel, can increase the cytotoxic and pro-apoptotic activity of the chemotherapeutic agent. It was noticed that the administration of PTX and CAPE changed the morphology of the cancer cells, reduced their viability and migration and also enhanced apoptosis. This synergistic effect was, however, dependent on the histological type of the ovarian cancer, the dose of CAPE and the duration of the experiment. The OV7 serous ovarian carcinoma cells were the most sensitive to the combined action of PTX + CAPE. The simultaneous administration of paclitaxel and CAPE to the HTB76 and CRL152 lines did not increase the drug’s anti-tumor effect. However, in all the tested lines, it was shown that the application of CAPE slowed down cell migration.

This experiment, which was conducted on commercially available cell lines, has its limitations. The defined phenotype and genotype of the tested lines as well as the culturing conditions may have affected the results obtained. In order to improve the quality of the experiment, it would be worth translating the in vitro model into research on animals or even using human material. Experiments conducted on a dissected cell line do not reflect the complexity of the processes taking place in a living organism. It is also necessary to take into account the metabolism of the tested compounds in the human body—the possibility of achieving effective doses in the tumor environment requires many studies. The mechanism of action of the tested compound still requires a lot of research.

An explanation of the mechanism of apoptosis induction in the cells treated with PTX+CAPE was taken into consideration when planning the next step of the experiment. It will be investigated whether the tested compounds activate caspases pathways and what is the role of mitochondria and pro-apoptotic proteins (e.g., TNF-α, sFasL, Bax and p53) in the induction of programmed cell death. The type of cell death induced by the tested compounds will also be investigated using electron microscopy techniques. Furthermore, an explanation as to why non-serous ovarian cancer cells are resistant to CAPE is necessary. It may be interesting to investigate the role of ABC membrane transporters (ATP-*binding cassette transporters*) that are responsible for the removal of chemotherapeutic agents from cells. Perhaps it will also be possible to lower the effective doses of the compounds tested so that the treatment of ovarian cancer can become even more effective and safe.

## Data Availability

The research data may be made available after contacting the corresponding author.

## Sample Availability

Not available.
